# Mothers’ and fathers’ engagement in math activities with their toddler sons and daughters: The moderating role of parental math beliefs

**DOI:** 10.3389/fpsyg.2023.1124056

**Published:** 2023-03-13

**Authors:** Alex M. Silver, Yu Chen, Darcy K. Smith, Catherine S. Tamis-LeMonda, Natasha Cabrera, Melissa E. Libertus

**Affiliations:** ^1^Department of Psychology, Learning Research and Development Center, University of Pittsburgh, Pittsburgh, PA, United States; ^2^Department of Human Development and Quantitative Methodology, University of Maryland, College Park, MD, United States; ^3^Department of Applied Psychology, Steinhardt School of Culture, Education and Human Development, New York University, New York, NY, United States

**Keywords:** home numeracy, math activities, gender roles, toddlers, math beliefs, fathers

## Abstract

Parents’ beliefs about the importance of math predicts their math engagement with their children. However, most work focuses on mothers’ math engagement with preschool- and school-aged children, leaving gaps in knowledge about fathers and the experiences of toddlers. We examined differences in mothers’ and fathers’ (*N* = 94) engagement in math- and non-math activities with their two-year-old girls and boys. Parents reported their beliefs about the importance of math and literacy for young children and their frequency of home learning activities. Parents of sons did not differ in their engagement in math activities from parents of daughters. Mothers reported engaging more frequently in math activities with their toddlers than fathers did, but the difference reduced when parents endorsed stronger beliefs about the importance of math for children. Even at very early ages, children experience vastly different opportunities to learn math in the home, with math-related experiences being shaped by both parent gender and parents’ beliefs.

## Introduction

Expectancy-Value Theory emphasizes connections among individuals’ values, expectations, and behaviors (Wigfield and Eccles, 2000). For example, as parents’ value for an activity increases, or the more they expect their child to enjoy, benefit, or succeed in a domain, the more frequently they should engage in that activity with their child. However, values and expectations do not emerge in a vacuum. Many factors affect parents’ values and expectations, including their beliefs around gender such as what skills girls or boys should learn and what activities mothers and fathers should engage in with their children. In this study, we examine parent–child math-related activities under the framework of Expectancy-Value Theory and consider how children’s and parents’ gender shape toddlers’ home engagement in math.

Mathematics provides an ideal domain for examining the role of parents’ expectations and attitudes, particularly in light of gender disparities in engagement. Gendered beliefs about math include stereotypes that math is a male-dominated domain (see Frost et al., 1994; Nosek et al., 2002) and that math requires innate brilliance (much more frequently attributed to males; see Chestnut et al., 2018). Adults’ math-gender stereotypes predict their expectations and values for boys’ and girls’ math achievement (see Eccles et al., 1990; Gunderson et al., 2012). Furthermore, parents’ gendered math attitudes and beliefs are associated with their children’s endorsement of gendered math attitudes and beliefs (e.g., Tenenbaum and Leaper, 2002; Hildebrand et al., 2022). Critically, by early-to mid-elementary school, children’s own math attitudes and beliefs are associated with their math achievement (see Levine and Pantoja, 2021).

### Why study math engagement in toddlers?

Math is a fundamental skill related to career choice, employment and income, and health and financial decision-making (Trusty et al., 2000; Currie and Thomas, 2001; Reyna and Brainerd, 2007; Agarwal and Mazumder, 2013). Individual differences in math performance emerge in early childhood (Starkey and Klein, 1992; Jordan et al., 2006) and predict children’s later math achievement and educational attainment throughout the school years and into adulthood (Duncan et al., 2007; Jordan et al., 2009; Siegler et al., 2012; Nguyen et al., 2016). Given the importance of math skills for daily life, much attention has been paid to identifying factors related to individual differences in early math achievement. Many contributing factors, including genetics (Hart et al., 2009) and social and environmental influences contribute to variability in early math performance (Jordan and Levine, 2009; Silver and Libertus, 2022).

Children’s home environment is a key influence that has received considerable attention, in particular, the extent to which parents engage in math-related activities with their children (Mutaf-Yildiz et al., 2020; Daucourt et al., 2021). Frequent home math activities, such as measuring ingredients while cooking or playing board games with dice or spinners, support children’s math performance (Blevins-Knabe and Musun-Miller, 1996; LeFevre et al., 2009; Kleemans et al., 2012; Niklas and Schneider, 2013; Ramani et al., 2015; Huntsinger et al., 2016; Mutaf Yildiz et al., 2018). However, relations are not always replicated (see Elliott and Bachman, 2018; Hornburg et al., 2021), suggesting that associations are complex and may depend on factors such as activity type (e.g., differences between formal, direct activities like doing number flashcards and informal, indirect activities like talking about money while shopping; Skwarchuk, 2009; DeFlorio and Beliakoff, 2014; Missall et al., 2014; Girard et al., 2021; Leyva et al., 2021), the quality of parent–child interactions while engaging in math activities (Elliott and Bachman, 2018), and children’s age (Thompson et al., 2017). Nonetheless, meta-analyses and systematic reviews of the literature suggest that home math engagement is helpful for children’s math performance, especially in early childhood (see Dunst et al., 2017; Mutaf-Yildiz et al., 2020; Daucourt et al., 2021). Investigating the factors that predict parental engagement in math activities with young children may therefore advance an understanding of how to support children’s early math development.

Previous work has focused primarily on factors related to variability in home math engagement in preschool-and school-aged children, with minimal attention to factors that contribute to home math engagement with infants and toddlers. However, variations in foundational number skills already emerge in infancy (e.g., Libertus and Brannon, 2010; Starr et al., 2013). Given the benefits of math engagement for the development of math skills in preschoolers and older children (e.g., Daucourt et al., 2021), further work is needed to understand how and why parents engage in math activities with younger children. Here, we describe parents’ math activities with their toddlers. We focus on child and parent characteristics found to be associated with parents’ engagement in general learning activities with toddlers and factors found to be associated with parents’ engagement in math activities with preschool-and school-aged children in prior studies.

### Parents’ home math activities with sons and daughters

We examined characteristics associated with differences in parents’ general engagement with toddlers to identify if similar relations apply to math engagement. One such factor is children’s gender, which has been studied extensively in other domains. The frequency with which parents engage in different types of home activities often differs for sons and daughters (see Morawska, 2020 for review). As early as infancy, parents hold different beliefs about the appropriate activities for boys and girls and tend to engage their sons in more physical play activities and daughters in more literacy activities (Leavell et al., 2011; Kroll et al., 2016; Dinkel and Snyder, 2020).

However, previous studies present conflicting results on parents’ math-specific engagement with sons and daughters. Some find that parents are more inclined to engage in math activities with their sons than with their daughters (Chang et al., 2011; Hart et al., 2016), whereas other studies indicate the reverse (Blevins-Knabe and Musun-Miller, 1996; Jacobs and Bleeker, 2004; del Río et al., 2017), or find no association between child gender and math engagement at home (Jordan et al., 2006; De Keyser et al., 2020; Zippert and Rittle-Johnson, 2020). Given the limited number of studies on the topic, and inconsistent findings, further inquiry into associations between child gender and math engagement at home is warranted.

### Mothers’ and fathers’ math engagement with children

Existing research on parents’ math engagement focuses on mothers (Blevins-Knabe and Musun-Miller, 1996; Jacobs and Bleeker, 2004; Byrnes and Wasik, 2009; del Río et al., 2017; De Keyser et al., 2020; Thippana et al., 2020; Zippert and Rittle-Johnson, 2020), pointing to the need to understand similarities and differences in how mothers and fathers engage their daughters and sons in math.

Mothers and fathers exhibit both similarities and differences in their style, quality, and frequency of engagement with young children in various activities, such as caregiving, reading, language input, and general cognitive stimulation activities, and father involvement uniquely relates to behaviors and developing skills in children after controlling for mothers’ involvement (Laflamme et al., 2002; Duursma et al., 2008; Baker, 2013; Duursma, 2014; Varghese and Wachen, 2015; Rolle et al., 2019; Cabrera et al., 2020). Mothers and fathers differ in how often they engage in literacy activities with their toddlers and how they read to them (e.g., Malin et al., 2014; Cabrera et al., 2020). Specifically, although mothers tend to engage more frequently in literacy activities (e.g., Burgess, 2010; Malin et al., 2014), fathers tend to use more complex and challenging language with their children (Ely et al., 1995; Rowe et al., 2004; Malin et al., 2014). Although research exists on differences in mothers’ and fathers’ talk and involvement with children about broader STEM topics (e.g., Crowley et al., 2001; Eccles, 2015), comparison of mothers’ and fathers’ math-specific engagement with children has received less attention.

Prior work comparing fathers’ and mothers’ involvement in math activities is considerably scarce and has focused exclusively on preschool-and school-aged children (e.g., Ramani et al., 2015; Elliott et al., 2017; Silver et al., 2020; Thippana et al., 2020). The handful of studies that have examined fathers’ home math-related engagement (focused on preschool-and school-aged children from different socioeconomic and cultural backgrounds) yield inconsistent results (Jacobs and Bleeker, 2004; Foster et al., 2016; Hart et al., 2016; del Río et al., 2017, 2019). Findings from two studies indicate that mothers may be more involved than fathers in math activities with their preschool-and kindergarten-aged children at home (Foster et al., 2016; del Río et al., 2019). However, others find no differences in mothers’ and fathers’ math engagement with kindergarten and school-aged children (Jacobs and Bleeker, 2004; del Río et al., 2017). Conflicting findings may be due to differences across studies in the types of math activities measured: In one study, mothers reported engaging in more numeracy activities than did fathers, but fathers reported engaging more frequently in overall home math activities (i.e., an overall composite of numeracy activities and spatial activities, such as drawing maps and measuring length and width) relative to mothers (Hart et al., 2016).

Inconsistent results across studies may be explained by differences in children’s age, other sample characteristics such as socioeconomic background, or the type of math activities measured. Even less is known about children’s engagement in math activities with their mothers and fathers during toddlerhood, the focus of this investigation.

### Parents’ math beliefs and math engagement

Mothers and fathers have been found to differ in math-related beliefs regarding sons and daughters (see Waters et al., 2022) in ways that may affect their math engagement. In particular, multiple types of math beliefs are found to influence parents’ engagement with preschool-and school-aged children, including parents’ perceptions of their role in their child’s math learning (Stipek et al., 1992; DeFlorio and Beliakoff, 2014; Sonnenschein et al., 2016), and beliefs about the importance of various academic subjects, including math (Cannon and Ginsburg, 2008; LeFevre et al., 2009; Puccioni, 2014).

Parents who hold strong beliefs about the importance of math for children (i.e., that math is an important skill for young children to learn) report engaging in frequent math-related activities with their preschool-and school-aged children (Musun-Miller and Blevins-Knabe, 1998; Cannon and Ginsburg, 2008; LeFevre et al., 2009; Sonnenschein et al., 2012; Muenks et al., 2015; Zippert and Ramani, 2017; Silver et al., 2021). Notably, these beliefs about the importance of math buffer against the negative consequences of math anxiety on parents’ engagement in math with their preschool-aged children (Silver et al., 2021).

However, most previous work focused on the math-related beliefs of parents of preschool-and school-aged children. Studies that targeted beliefs of parents with infants and toddlers largely examined parents’ beliefs about parenting, such as their role in co-parenting, the importance of play, and their goals for children (e.g., Coleman and Karraker, 2003; Rowe and Casillas, 2011; Favez et al., 2015; Manz and Bracaliello, 2016), and uniformly find positive associations between beliefs and engagement. It remains unknown whether parents’ math-specific beliefs, and in particular their beliefs about the importance of math, predict their math engagement with toddlers.

### The current study

We sought to identify whether child and parent gender and parents’ beliefs about the importance of math relate to parental engagement in math activities with toddlers. We first explore whether children’s and/or parents’ gender relate to differences in home math activities. Based on inconsistent prior findings, we were uncertain about the role of children’s and parents’ gender in parents’ math activities. Second, we investigate associations between parents’ beliefs about the importance of math for young children and their home math activities. We expected these math beliefs to positively relate to parents’ engagement in math activities with their children, based on prior work with parents of older children (Musun-Miller and Blevins-Knabe, 1998; Cannon and Ginsburg, 2008; LeFevre et al., 2009; Sonnenschein et al., 2012; Muenks et al., 2015; Zippert and Ramani, 2017; Silver et al., 2021), and in line with the idea that strong beliefs about the importance of math increase the value parents place on math engagement with their children (Wigfield and Eccles, 2000). Next, we examine whether parents’ beliefs about the importance of math moderate the effects of children’s and parents’ gender on parents’ math activies. We expected associations between children’s and parents’ gender and parents’ frequency of engaging in math activities to be moderated by parents’ beliefs about math, such that stronger beliefs about the importance of math might buffer (i.e., reduce) gender differences in math activities. Prior work shows that parents’ positive beliefs about children’s abilities and the importance of school can buffer against children’s low school attitudes, expectations, and performance (Wigfield and Gladstone, 2019), and specifically that parents’ beliefs about the importance of math buffer against the negative influence of parental math anxiety (Silver et al., 2021).

Finally, we examine the robustness and domain-specificity of these effects to determine whether associations are specific to math or apply to parental engagement broadly. To test specificity of associations, we controlled for other potentially confounding family characteristics, including children’s age, parents’ education, parents’ language, parents’ beliefs about the importance of domains other than math, and parents’ engagement in non-math activities. Although children were all 2 years of age, we controlled for children’s age given prior findings that parents may change their engagement in math activities as children develop (e.g., Thompson et al., 2017; Daucourt et al., 2021). We controlled for parents’ education and language to ensure that any differences in math activities were not due to socioeconomic or cultural assimilation differences between families (see Vigdor, 2009; Eason et al., 2022). We controlled for parents’ beliefs about the importance of literacy and engagement in non-math activities to test whether associations were specific to parents’ beliefs about the importance of math and math activities, rather than beliefs about the importance of academic skills generally or engagement in learning activities broadly. Finally, to further probe the specificity of these associations, we ran follow-up analyses on parents’ beliefs about the importance of literacy and non-math activities.

## Method

### Participants

Data were drawn from a multi-site study on how mothers and fathers from ethnically diverse two-parent households support their two-year-old children’s acquisition of academic skills. Participants were 94 parents of toddlers (52 mothers, 42 fathers; 40 families had both the child’s mother and father participate) from the New York City, New York (26 parents), Pittsburgh, Pennsylvania (28 parents), and College Park, Maryland (40 parents) metropolitan areas of the United States. An additional four parents participated in the study but did not complete all measures and were not included in analyses. Parents were Hispanic/Latino (65%) and White, non-Hispanic/Latino (35%). Half indicated a preference to participate in English (*n* = 47) and half chose to participate in all tasks in Spanish (*n* = 47). Participants averaged 13.10 years of education (SD = 3.77 years; range from 4 years to 17 years).

### Procedure

Participants were recruited *via* flyers, online postings, and in-person recruitment at local daycare centers in three metropolitan areas of the eastern United States. Due to the broader aims of the study, families were eligible to participate if both parents lived at home with the child, had obtained no more than a Bachelor’s degree, spoke only English and/or Spanish, and were either White, non-Hispanic/Latino or Hispanic/Latino. At each site, mothers and fathers and their children participated in two home visits. Parents were told that the study focused on how parents play with their young children and support toddlers’ development in the home, and they were not told that the focus of the study was on math. The data used for this project are drawn from a self-report questionnaire that all parents completed with researchers during the home visit, describing their frequency of engaging in learning activities with their child, their attitudes, beliefs, and anxiety about engaging in various academic activities, and demographic information about their family. Parents also completed math and spatial assessments, a non-symbolic number comparison task, and participated in semi-structured observations with their child. These measures were not the focus of this study, and so are not discussed further. Each parent received $50 for participation.

### Measures

#### Parents’ home learning activities

Each parent reported the frequency of home learning activities they engage in with their child. The full list of items can be found in the Supplemental Material. Parents were asked to indicate how often in the past month they had participated in listed activities (e.g., 11 math activities such as “Counting objects”; 9 non-math activities such as “Coloring, painting, writing” or “Identifying names of written alphabet letters”) with their child on a scale from 1 (“Did not occur”) to 5 (“Almost daily”), with additional options to indicate whether the listed activity was not appropriate for their child due to age or was not appropriate for their family because they did not own the items necessary to engage in the activity (which was scored as “NA”). Responses for the 11 math-related items were averaged to create a math activities score, and responses for the 9 non-math items were averaged to create a non-math activities score.

#### Parents’ beliefs about the importance of math and literacy for young children

Each parent reported their beliefs about the importance of math and literacy for young children using the Benchmarks Survey from the Home Numeracy Questionnaire (LeFevre et al., 2009). The full list of items can be found in the Supplemental Material. They were asked, “In your opinion, how important is it for children to reach the following benchmarks prior to entering kindergarten?” on a scale from 1 (“Not at all important”) to 5 (“Very important”). Items included parents’ beliefs about the importance of five math skills (e.g., “Count to 100″) and four reading and writing skills (e.g., “Print alphabet letters”). Responses to the five math items were averaged to create a belief about the importance of math score, and responses to the four literacy items were averaged to create a belief about the importance of literacy score.

#### Children’s and parents’ gender

Child and parent gender were coded using effects coding (where female = 0.5, male = −0.5).

#### Family demographic information

Parents reported their child’s birthdate, which was used to calculate the child’s age in months on the date of testing. In addition, each parent reported how many years of school they had completed, and the language that they preferred to use for testing.

### Data analysis and model fitting

Due to the clustering present in our data (where individual parents are nested within families, and families are nested within three sites of data collection), mixed effects models predicting the frequency of parents’ engagement in math activities with their children were tested and compared using the *lme4* and *lmertest* packages in R (Bates et al., 2007; Kuznetsova et al., 2017). All tested models included random effects for family and site, and prior to analysis we standardized all variables to allow for ease of interpretation of results. In a series of hierarchical mixed effects models, we predicted parents’ engagement in math activities.

In Model 1, we predicted parents’ engagement in math activities from fixed effects of children’s gender, parents’ gender, and parents’ beliefs about the importance of math. In Model 2, we used the same fixed effects as in Model 1, with the addition of an interaction between children’s gender and parents’ beliefs about the importance of math. In Model 3, we used the same fixed effects as in Model 1, with the addition of an interaction between parents’ gender and parents’ beliefs about the importance of math.

#### Follow-up models testing for robustness and domain-specificity

For any significant interactions found in Models 2 or 3, we ran follow-up analyses controlling for possible confounds (Step 4), testing robustness of the results (Step 5), and examining the domain-specificity of the interactions (Steps 6 and 7).

To control for possible confounds of family demographic characteristics, in Step 4 we added fixed effects of children’s age, parents’ education, and parents’ language used. As a particularly stringent test of the robustness of our results, in Step 5 we added fixed effects of parents’ non-math activity engagement and parents’ beliefs about the importance of literacy.

Finally, in Steps 6 and 7 we explored the domain-specificity of associations (i.e., whether associations were characteristic of parents’ activities with their toddlers broadly or specific to their math activities). Specifically, in Step 6, for significant interactions in Models 2 or 3, we first tested a model predicting parents’ engagement in non-math activities from those same predictors and controlling for math activities. A significant interaction in predicting non-math activities would indicate that associations are not specific to math. In contrast, a non-significant interaction would suggest that the association is specific only to math activities.

In Step 7 we tested a second follow-up model predicting parents’ engagement in math activities from the same predictors but using parents’ beliefs about the importance of literacy in the interaction (instead of their beliefs about the importance of math). A significant interaction between parents’ beliefs about the importance of literacy and children’s or parents’ gender would indicate a domain-general association (as parental beliefs about the importance of skills across domains moderate associations of gender with math engagement). However, a non-significant interaction would suggest that the association is specific to beliefs about the importance of math specifically.

#### Model fitting

This dataset included data at three different levels, such that Level 1 is the individual parent participant, Level 2 is the family from which each parent comes, and Level 3 is the site from which each family was recruited and tested. In all models, random effects included intercepts for each family and each data collection site to account for clustering within families and within geographic sites of data collection. The maximal models were initially tested but failed to converge. To maintain the maximal random effects structure, the correlation parameters were removed from the models. This led the models to converge but they remain overfitted as indicated by a “singular fit” warning. To further reduce model complexity, the random slopes for children’s age, parents’ years of education, parents’ frequency of engaging in non-math activities, parents’ beliefs about the importance of math and parents’ beliefs about the importance of literacy (which had all been included for both family and site to account for potential differences in how the fixed effects may relate to math activities within families and sites) were removed. Model comparison indicated that models not containing random slopes better fit the data [with lower Akaike Information Criterion (AIC) and Bayesian Information Criterion (BIC)], and the statistical significance of all main effects and interactions remained consistent in models with the inclusion and exclusion of the random slopes. Therefore, for parsimony, the final models did not include the random slopes or correlations.

## Results

Descriptive statistics of parents’ math activities with their toddlers are presented in Table 1. Parents engaged in math activities with their toddlers on average about once a week (Mean = 3.09; Median = 3.18) with wide variability (ranging from never to almost daily). Over 54% of parents reported engaging in math activities more than once per week. Parents reported engaging more frequently in non-math activities (Mean = 3.52, corresponding to between once a week and a few times a week; Median = 3.67) than math activities, *t*(93) = −6.56, *p* < 0.001. Over 76% of parents reported engaging in non-math activities more than once per week, and more than 77% of parents reported more frequent non-math activities than math activities. Item-level descriptive statistics for the home learning activities measure can be found in Table 2.

**Table 1:** Descriptive statistics for study variables.

Variable	Overall *M* (SD)	Overall Range	Mother *M* (SD)	Father *M* (SD)	Child Female *M* (SD)	Child Male *M* (SD)	Spanish *M* (SD)	English *M* (SD)
Math activities	3.09 (0.82)	1.00–4.57	3.25 (0.67)	2.88 (0.94)	3.01 (0.83)	3.16 (0.80)	3.03 (0.87)	3.14 (0.76)
Math beliefs	3.68 (0.88)	1.20–5.00	3.60 (0.89)	3.79 (0.87)	3.74 (0.83)	3.62 (0.93)	3.80 (0.92)	3.56 (0.83)
Literacy beliefs	4.16 (0.84)	1.00–5.00	4.15 (0.87)	4.18 (0.80)	4.19 (0.74)	4.14 (0.93)	4.21 (0.79)	4.11 (0.89)
Child age (months)	30.78 (3.58)	24.26–36.39	30.60 (3.64)	31.00 (3.53)	31.10 (3.94)	30.40 (3.19)	30.20 (3.50)	31.40 (3.58)
Parent education (years)	13.10 (3.77)	4.00–17.00	13.10 (3.69)	13.10 (3.90)	13.10 (4.02)	13.10 (3.55)	11.70 (4.01)	14.50 (2.96)
Non-math activities	3.52 (0.75)	1.00–4.75	3.70 (0.66)	3.29 (0.80)	3.55 (0.75)	3.48 (0.76)	3.32 (0.83)	3.71 (0.61)
Variable	Overall *N*		Mother *N*	Father *N*	Child Female *N*	Child Male *N*	Spanish *N*	English *N*
Child gender Female	46		26	20	–	–	24	22
Child gender Male	48		26	22	–	–	23	25
Language Spanish	47		26	21	24	23	–	–
Language English	47		26	21	22	25	–	–

Overall *N* = 94 (Mother *N* = 52, Father *N* = 42; Child Gender Female *N* = 46, Child Gender Male *N* = 48; Language Used Spanish *N* = 47, Language Used English *N* = 47). Activities frequency could range from 1 (“did not occur”) to 5 (“almost daily”), and beliefs about the importance of skills could range from 1 (“not important”) to 5 (“very important”).

**Table 2:** Item-level descriptive statistics for home learning activities.

Home learning activity	*M* (SD)	Number of “My child is still too young for that” Responses	Number of “Do not have” Responses
Counting objects	4.13 (1.15)	0	0
Sorting things by size, color or shape	3.33 (1.25)	0	0
Counting down	2.45 (1.45)	6	0
Identifying names of written numbers	3.06 (1.54)	4	0
Picking up sticks, objects, etc.	4.33 (1.18)	1	0
Buttoning buttons	2.37 (1.41)	11	0
Movement songs (i.e., Itsy Bitsy Spider)	4.16 (1.26)	4	0
Coloring, painting, writing	3.97 (1.21)	0	0
Identifying names of written alphabet letters	3.48 (1.41)	6	0
Identifying sounds of alphabet letters	3.09 (1.45)	7	0
Making music	3.72 (1.44)	0	0
Playing with number fridge magnets	2.70 (1.59)	0	31
Putting pegs in a board or shapes into holes	3.26 (1.38)	0	20
Playing with puzzles	3.14 (1.32)	0	11
Building with blocks or construction sets (Duplo, Megablocks, etc.)	3.87 (1.20)	0	12
Playing with “Playdoh,” dough, or clay	3.05 (1.47)	0	15
Using number activity books (like connect-the-dots)	2.53 (1.43)	0	13
Playing board games with numbers	2.03 (1.26)	0	29
Reading books that teach simple shapes like squares, circles, and triangles	3.12 (1.39)	0	5
Recite nursery rhymes (such a “Mother Goose”) or read other rhyming books	3.27 (1.49)	0	12

Frequency of activities ranged from 1 (“Did not occur”) to 5 (“Almost daily”). Parents were given options to indicate if “My child is still too young for that” or if they “Do not have” the physical materials to participate.

Parents’ beliefs about the importance of math for young children also varied widely, with parents reporting on average that they believed math was moderately to quite important (Mean = 3.68; Median = 3.80), with beliefs ranging from not at all important to very important. Over 34% of parents reported that math was quite important or very important. Parents’ beliefs about the importance of literacy for young children (Mean = 4.16, corresponding to between quite important and very important; Median = 4.25) were significantly higher than their beliefs about the importance of math, *t*(93) = −7.27, *p* < 0.001. Over 54% of parents reported beliefs that literacy was quite important or very important, and over 85% of parents reported higher beliefs about the importance of literacy than about the importance of math.

We next asked whether parents’ frequency of engaging in math activities differed with sons and daughters or for mothers and fathers, and whether parents’ beliefs about the importance of math moderated these associations (results from Models 1–3 can be found in Table 3). In all models we included random effects of family and site, which together accounted for 18.1% of the variance in parents’ engagement in math activities. Model 1 tested fixed effects of children’s gender, parents’ gender, and parents’ beliefs about the importance of math on parents’ math activities, and explained 7.4% of the variance in math activities. Parents of sons and parents of daughters did not differ in their reported math activities, but overall mothers engaged in significantly more frequent math activities than fathers did (*B =* 0.40, 95% CI [0.11, 0.70], *p* = 0.011). Contrary to hypotheses, we found no significant main effect of parents’ beliefs about the importance of math on math activities.

**Table 3:** Mixed effects models predicting parents’ engagement in math activities.

	Model 1		Model 2		Model 3	
Fixed effect	*B*	95% CI	*B*	95% CI	*B*	95% CI
Intercept	3.06**	[2.81, 3.31]	3.06**	[2.82, 3.31]	3.04**	[2.75, 3.34]
Child gender	−0.12	[−0.46, 0.22]	−0.12	[−0.47, 0.23]	−0.06	[−0.41, 0.29]
Parent gender	0.40*	[0.11, 0.70]	0.40*	[0.11, 0.71]	0.41**	[0.13, 0.70]
Math beliefs	0.11	[−0.06, 0.27]	0.10	[−0.06, 0.27]	0.12	[−0.05, 0.28]
Child gender X Math beliefs	–	–	−0.04	[−0.36, 029]	–	–
Parent gender X Math beliefs	–	–	–	–	−0.32*	[−0.63, 0.00]
Random effect	SD		SD		SD	
Family intercept	0. 32		0.32		0.37	
Site intercept	0.16		0.16		0.21	
Residual	0.72		0.72		0.68	

*p < 0.05; **p < 0.01.

We next tested whether parents’ beliefs about the importance of math might moderate associations between children’s or parents’ gender and parents’ math activity engagement. Model 2 tested whether parents’ beliefs about the importance of math moderate the association between children’s gender and parents’ math activities but found no significant interaction. In Model 3 a significant interaction was found between parents’ beliefs about the importance of math and parents’ gender (*B* = −0.31, 95% CI [−0.63, 0.00]), such that the effect of parents’ gender (where mothers engage in more frequent math activities than fathers) is reduced when parents hold strong beliefs about the importance of math for young children. Model 3 accounted for significantly more variance in math activities than Model 1 (ΔR^2^ = 0.03, 95% CI [0.02, 0.05], *p* < 0.001), and was a marginally significantly better fit of the data than Model 1, *χ*^2^(1) = 3.27, *p* = 0.07. Critically, the pattern of main effects from Model 1 remained similar in Model 3, with a significant effect of parents’ gender (*B* = 0.41, 95% CI [0.13, 0.70], *p* = 0.007) and no main effect of children’s gender and parents’ beliefs about the importance of math.

**Figure 1:**
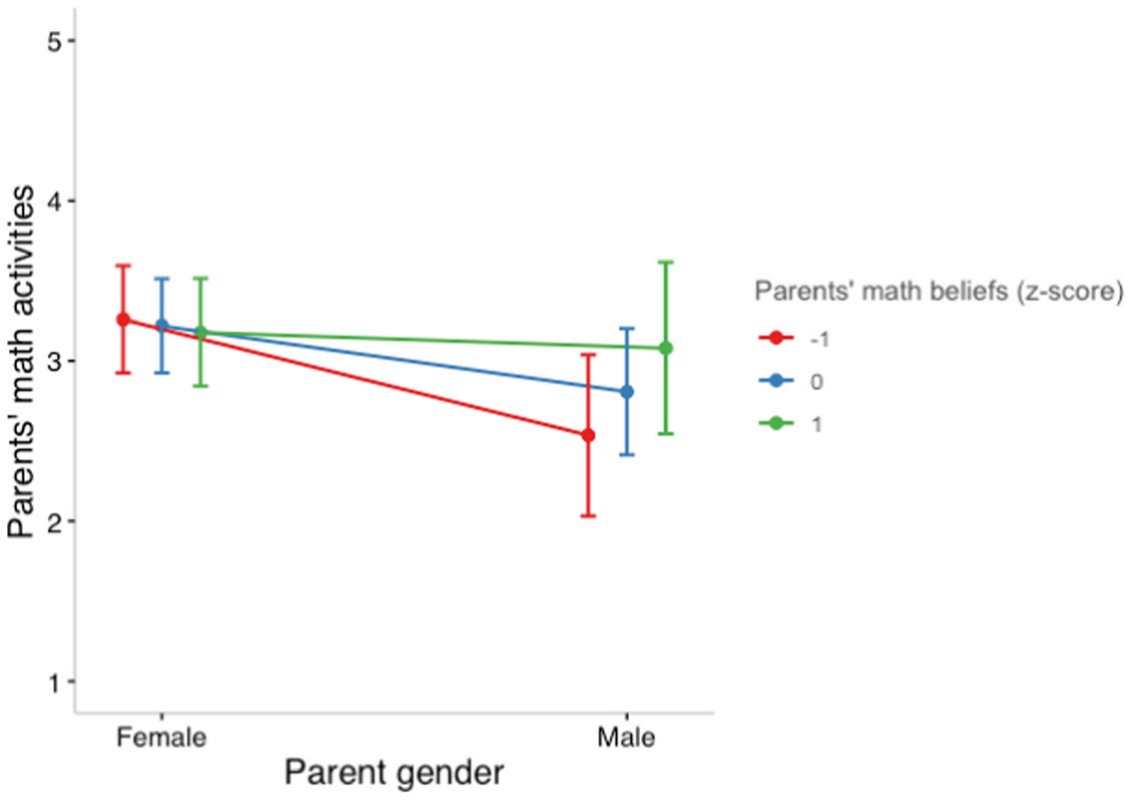
Interaction between parents’ gender and parents’ beliefs about the importance of math for young children predicting parents’ math activities. The frequency of parents’ home math activities ranged from 1 (“Did not occur”) to 5 (“Almost daily”).

Given the significant interaction between parents’ beliefs about the importance of math and parents’ gender in Model 3, we next tested the robustness of results in a series of follow-up analyses. In Model 4, we used the same predictors as in Model 3 and included fixed effects of children’s age, parents’ education, and parents’ language as controls. Parents’ gender continued to predict math activities (*B* = 0.40, 95% CI [0.11, 0.69], *p* = 0.010), and the interaction between beliefs about the importance of math and parents’ gender also remained significant (*B* = −0.35, 95% CI [−0.68, −0.02], *p* = 0.039) even with the addition of these control variables. In Model 5 we added fixed effects of parents’ non-math activities and parents’ beliefs about the importance of literacy to Model 4 for a final stringent robustness check. Model 5 explained 49.4% of the variance in parents’ math activities and was a significantly better fit than any of the previously tested models. Although the main effect of parents’ gender was no longer significant in Model 5, even with the addition of these stringent control variables the interaction between parents’ beliefs about the importance of math and parents’ gender remained significant (*B* = −0.32, 95% CI [−0.56, −0.07], *p* = 0.014; see Figure 1). Results from Models 4 and 5 can be found in Table 4.

**Table 4:** Follow-up mixed effects models predicting parents’ engagement in math activities with additional control variables.

	Model 4		Model 5	
Fixed effect	*B*	95% CI	*B*	95% CI
Intercept	3.04*	[2.82, 3.26]	3.06*	[2.88, 3.24]
Child gender	−0.07	[−0.42, 0.28]	−0.13	[−0.39, 0.13]
Parent gender	0.40*	[0.11, 0.69]	0.08	[−0.15, 0.32]
Math beliefs	0.13	[−0.03, 0.30]	0.13	[−0.06, 0.31]
Parent gender X math beliefs	−0.35*	[−0.68, −0.02]	−0.32*	[−0.56, −0.07]
Child age	−0.12	[−0.29, 0.06]	−0.12	[−0.25, 0.02]
Parent education	0.09	[−0.09, 0.27]	−0.01	[−0.14, 0.13]
Language used	0.13	[−0.26, 0.52]	−0.11	[−0.42, 0.68]
Non-math activities	–	–	0.55***	[0.42, 0.68]
Literacy beliefs	–	–	−0.12	[−0.29, 0.06]
Random effect	SD		SD	
Family intercept	0.35		0.22	
Site intercept	0.12		0.11	
Residual	0.70		0.54	

*p < 0.05; **p < 0.01; ***p < 0.001.

To explore domain-specificity of the significant interaction between parents’ beliefs about the importance of math and parents’ gender, we tested follow-up Models 6 and 7. Model 6 predicted parents’ engagement in non-math activities from the same set of predictors as Model 5. The interaction between parents’ beliefs about the importance of math and parents’ gender did not predict parents’ non-math activities (*B* = 0.17, 95% CI [−0.6, 0.40], *p* = 0.154). Finally, Model 7 predicted parents’ engagement in math activities from the same set of predictors as Model 5, but with an interaction between parents’ beliefs about the importance of literacy (rather than beliefs about the importance of math) and parents’ gender. The interaction between parents’ beliefs about the importance of literacy and parents’ gender was not significant in predicting parents’ math activities (*B* = −0.22, 95% CI [−0.48, 0.03], *p* = 0.087). The results of Models 6 and 7 (which can be found in Table 5) suggest that the interaction between parents’ beliefs about the importance of math for young children and parents’ gender are domain-specific to math activities and beliefs about the importance of math.

**Table 5:** Follow-up mixed effects models testing domain-specificity of results predicting parents’ engagement in non-math activities (Model 6) and parents’ engagement in math activities (Model 7).

	Model 6		Model 7	
Fixed effect	*B*	95% CI	*B*	95% CI
Intercept	3.52*	[3.41, 3.62]	3.08*	[2.87, 3.29]
Child gender	0.13	[−0.09, 0.34]	−0.15	[−0.41, 0.11]
Parent gender	0.19	[−0.03, 0.41]	0.07	[−0.17, 0.32]
Math beliefs	−0.02	[−0.18, 0.14]	0.07	[−0.12, 0.26]
Parent gender X math beliefs	0.17	[−0.06, 0.40]	–	–
Child age	0.06	[−0.05, 0.17]	−0.11	[−0.24, 0.03]
Parent education	0.07	[−0.05, 0.19]	−0.03	[−0.17, 0.10]
Language used	0.24*	[0.00, 0.47]	−0.16	[−0.48, 0.16]
Non-math activities	–	–	0.55***	[0.42, 0.69]
Literacy beliefs	0.10	[−0.06, 0.26]	−0.06	[−0.25, 0.12]
Math activities	0.48***	[0.37, 0.60]	–	–
Parent gender X literacy beliefs	–	–	−0.22	[−0.48, 0.03]
Random effect	SD		SD	
Family intercept	0.00		0.22	
Site intercept	0.00		0.15	
Residual	0.00		0.55	

*p < 0.05; ***p < 0.001.

## Discussion

Parental engagement in math activities at home has been found to predict children’s math skills, but this work has primarily focused on preschool-and school-aged children (e.g., LeFevre et al., 2009; Mutaf-Yildiz et al., 2020; Daucourt et al., 2021). Here, we find that parents differ widely in their engagement in math activities with toddlers, and that parents’ beliefs about the importance of math and parents’ gender play a role in parents’ engagement in math activities with toddlers. Furthermore, we find that the effects of parents’ beliefs about the importance of math (in interaction with parent gender) are specific to the domain of math.

We found that the main effect of children’s gender was not significant. Instead, and in line with some other past work studying preschool-and school-aged children (Jordan et al., 2006; De Keyser et al., 2020; Zippert and Rittle-Johnson, 2020), parents did not differ in their math activities with 2-year-old sons and daughters. Similarly, although parents’ gender significantly predicted their math activities in some models, when controlling for parents’ beliefs about the importance of literacy skills and their engagement in non-math activities this main effect disappeared. Together with inconsistent findings in the literature (e.g., Blevins-Knabe and Musun-Miller, 1996; Jacobs and Bleeker, 2004; Chang et al., 2011; Foster et al., 2016; del Río et al., 2017, 2019; Thippana et al., 2020), our findings suggest the need for further inquiry into the specific contexts in which children’s and parents’ gender relate to math engagement.

Existing studies vary widely on the types of math engagement measured (e.g., math activities versus math talk), the ages of children involved (e.g., toddlers versus preschool-aged versus school-aged children), the methods of data collection (e.g., parent-report measures versus direct observations), the countries of origin for participants (e.g., Chile versus Belgium versus the United States), the demographics of the families involved (e.g., predominantly middle-to upper-income versus lower-income), the gender of parents involved in the study (e.g., predominantly mothers versus mothers and fathers), and the historical cohort of parents in the samples (e.g., 1970s versus 2010s). Therefore, conflicting results across studies are unsurprising, and point to the need to consider variables that may moderate associations between children’s and parents’ gender and parent–child math engagement.

Indeed, we find that parents’ beliefs about the importance of math moderated the effects of parent gender on math activities. Mothers and fathers differed in their engagement in math activities, but only in the presence of low parental beliefs about the importance of math for young children, such that mothers engaged in more frequent math activities than fathers did. When parents held strong beliefs about the importance of math, these gender differences reduced. Unmeasured parent beliefs may explain some of the inconsistent gender findings in the literature: If differences in math engagement by children’s and parents’ gender emerge only in some contexts (i.e., in the presence of particular parental math beliefs), samples in previous studies may have differed in their math beliefs.

### Parents’ beliefs about the importance of math for young children

Previous work with older children found that parents’ beliefs about the importance of math for their children related to their frequency of engagement in math activities (e.g., Musun-Miller and Blevins-Knabe, 1998; Cannon and Ginsburg, 2008; LeFevre et al., 2009; Sonnenschein et al., 2012; Muenks et al., 2015; Zippert and Ramani, 2017; Silver et al., 2021). Contrary to these findings, we did not find such an association for parents of toddlers. Perhaps parents of toddlers, whose children are still years away from beginning kindergarten and formal education, do not yet hold strong beliefs about the importance of math; as children begin formal schooling, parents may increase their beliefs about math’s importance. Future work on parents’ beliefs about the importance of math for young children of different ages may prove useful to test how child age may shape parent beliefs.

We further examined whether associations between parents’ beliefs about the importance of math and their engagement in math activities might differ based on children’s or parents’ gender. Along with a null effect of children’s gender, parents’ beliefs about the importance of math for young children did not moderate the effect of children’s gender on math engagement. Thus, parents of sons and parents of daughters were similar in their frequency of math activities, regardless of their beliefs about the importance of math. In contrast, the parent gender gap in math activities (in which mothers engaged in more frequent math activities than fathers) was reduced for parents with strong beliefs about the importance of math for young children. Interestingly, mothers engaged in similar frequencies of math activities regardless of their beliefs about the importance of math, whereas fathers with strong beliefs about the importance of math for young children engaged in more frequent math activities than fathers with less strong beliefs.

Why might this be? Prior research indicates that mothers are generally more involved in young children’s daily activities than fathers (Duursma, 2014; Cabrera et al., 2020). As a result, mothers may engage in fairly frequent math activities regardless of how important they believe math skills are, whereas fathers may be motivated to engage in such activities by strong beliefs that math skills are important for children. Along those lines, mothers and fathers may differ in the types of activities they engage in with their child (Hart et al., 2016). Formal activities may require explicit beliefs about the importance of engaging with and teaching children, whereas informal activities may not depend on such strong beliefs. Here, we combined across math activities (due to a limited number of items preventing subanalyses on formal and informal activities), but mothers and fathers may have engaged in qualitatively different activities. Moreover, other parent math beliefs not measured here may affect parents’ engagement in math activities. Future work should examine how these relations persist or change when controlling for other parental math beliefs.

Other types of math beliefs (beyond the importance of math) may relate to parents’ math engagement and moderate associations between children’s and parents’ gender and parents’ math engagement. Parents may vary in their beliefs about their children’s propensity to learn math; their views on their own role and responsibility in helping their children learn math; their expectations for what their children can learn at different ages; their views about appropriate developmental activities for children of specific ages; their beliefs about the fixedness or malleability of math ability; and their gender stereotypes. All not measured here, such beliefs may relate to parents’ engagement in math activities with toddlers and account for the different patterns of engagement we observe. Importantly, future work should expand an understanding of how a variety of math beliefs relate to parents’ math engagement with their children and potentially interact with parents’ and children’s gender, to help disentangle these effects. Furthermore, it will be crucial to understand when and where these parental beliefs originate and how they change through children’s development, and their consequences for parents’ math engagement.

### Limitations, conclusions and future directions

Several limitations merit discussion. Our sample, though diverse in educational background, comprised only White, non-Hispanic/Latino and Hispanic/Latino families. Although we saw no differences in parents’ math engagement based on the language they spoke (a measure of cultural assimilation; Vigdor, 2009), our findings may not extend to other populations in other contexts. Indeed, parents from different ethnic backgrounds differ in their beliefs and general engagement with their children (e.g., Suizzo, 2007; Keels, 2009), indicating a need for future work on similarities and differences in associations between children’s and parents’ gender, parents’ beliefs about the importance of math, and parent–child math engagement. Furthermore, concurrent associations examined here do not inform on causality. Longitudinal analyses are needed to examine how these relations change over time, and experimental work is needed to determine which types of math activities may specifically support which types of math skills in young children. Relatedly, future work may investigate whether the benefits that children receive from parental math engagement differ based on the gender of the parent involved.

Furthermore, we studied two-year-old toddlers, and observed associations may change with age. Additionally, parents’ engagement in math activities may be shaped by other factors not measured here, including, (but certainly not limited to) parents’ own math abilities, parents’ employment status, children’s enrollment in preschool, and the number of other children in the home. We included a control for parents’ engagement in non-math activities, which likely would be influenced by some of these factors as well, but future work examining these associations with the addition of critical covariates is warranted. Finally, our measures of parents’ beliefs and activities were drawn from self-report questionnaires. As such, the reports may be subject to reporter bias of over-or under-reporting of activities or beliefs. In addition, the math activity questionnaire was composed of only 11 items, which may not capture other math-related activities that parents and children may engage in, parents’ use of math talk and math engagement outside of the queried specifically math-related activities, the durations of the activities, and the quality of math content discussed during the activities (see Elliott and Bachman, 2018).

Nonetheless, findings suggest the importance of considering how parents’ and children’s gender shape parents’ beliefs and in turn their math engagement with toddlers. More generally, these results add to our understanding of the factors that relate to the home learning environment, showing that even at very young ages children are exposed to vastly different amounts of math support. Whether and how differences in home math engagement relate to toddlers’ early math skills, and how such findings might inform interventions around parents’ support of children’s early emerging math skills, are critical future directions.

## Data availability statement

The original contributions presented in the study are publicly available. This data can be found with DOI 10.17605/OSF.IO/35SVB here: https://osf.io/35svb/.

## Ethics statement

The studies involving human participants were reviewed and approved by University of Pittsburgh Institutional Review Board, University of Maryland Institutional Review Board, and New York University Institutional Review Board. Written informed consent to participate in this study was provided by the participants’ legal guardian/next of kin.

## Author contributions

AS performed the statistical analysis and wrote the first draft of the manuscript. All authors contributed to the conception, design of the study, manuscript revision, read, and approved the submitted version.

## Funding

This work was funded by the National Science Foundation (HRD1760844 to ML, HRD1760643 to NC, and HRD1761053 to CT-L). AS was supported by the National Institutes of Health under grant T32GM081760, and ML was supported by a Scholar Award from the James S. McDonnell Foundation.

## Conflict of interest

The authors declare that the research was conducted in the absence of any commercial or financial relationships that could be construed as a potential conflict of interest.

## Publisher’s note

All claims expressed in this article are solely those of the authors and do not necessarily represent those of their affiliated organizations, or those of the publisher, the editors and the reviewers. Any product that may be evaluated in this article, or claim that may be made by its manufacturer, is not guaranteed or endorsed by the publisher.
